# An efficient wave-optics framework for partially coherent pink X-ray beam simulation and analysis

**DOI:** 10.1107/S1600577526007034

**Published:** 2026-08-31

**Authors:** Shuo Wang, Han Xu, Liang Zhou, Ming Li

**Affiliations:** a Beijing Synchrotron Radiation Facility Institute of High Energy Physics, Chinese Academy of Sciences Beijing People’s Republic of China; b University of Chinese Academy of Sciences Beijing 100049 People’s Republic of China; Australian Synchrotron, Australia

**Keywords:** algorithm, X-ray simulation, high efficiency, mode decomposition, hierarchical incremental singular value decomposition

## Abstract

The advent of fourth-generation synchrotron radiation sources has enabled advanced coherent X-ray techniques. However, high-flux pink-beam operation introduces significant computational challenges for wave-optics-based coherence analysis. Conventional approaches, such as Monte Carlo sampling of the electron beam or direct diagonalization of the cross-spectral density, become computationally prohibitive when extended to broadband spectra. In this work, we develop an integrated computational framework that combines Monte Carlo brightness convolution for efficient wavefront matrix construction, hierarchical incremental singular value decomposition to overcome memory bottlenecks in coherent mode decomposition, and spectral–spatial decomposition to extract a global orthogonal basis for rapid broadband propagation. The reliability of the proposed method is validated through comparison with *Synchrotron Radiation Workshop* (*SRW*) simulations and experimental measurements performed at the HEPS Hard X-ray Coherent Scattering (HXCS) beamline. Furthermore, the framework is applied to analyze chromatic aberrations in compound refractive lens focusing systems, quantitatively evaluating focal-spot broadening and coherence degradation inherent to pink-beam operation. The proposed framework provides a practical and scalable tool for the design and optimization of high-flux pink-beam coherent experiments at modern synchrotron facilities.

## Abstract 2

This article proposes a new calculation method and framework for simulating a pink X-ray beam, significantly improving computational efficiency. By comparing it with the experimental data from the beamline, the simulation method is consistent with the actual situation.

## 1. Introduction

### 1.1. Monte Carlo based brightness convolution for wavefront matrix construction

Fourth-generation synchrotron radiation sources (Hettel, 2014 ▸), such as ESRF-EBS (Raimondi, 2016 ▸), APS-U (Calvey *et al.*, 2025 ▸) and HEPS (Jiao *et al.*, 2018 ▸), deliver X-ray beams characterized by high transverse coherence. This enhanced coherence enables advanced coherent X-ray techniques (Girelli *et al.*, 2021 ▸; Lehmkühler *et al.*, 2021 ▸; Miao, 2025 ▸), including X-ray photon correlation spectroscopy, coherent diffraction imaging and ptychography. In practice, however, coherent X-ray experiments are often photon-limited, particularly in time-resolved studies (Yildirim *et al.*, 2025 ▸) and the investigations of weakly scattering materials (Meents *et al.*, 2017 ▸). Therefore, employing X-ray beams with a finite energy bandwidth (commonly referred to as pink X-ray beams) is essential for maximizing photon flux and achieving practical measurement times. For an undulator source followed by a crystal monochromator, the resulting monochromatic X-ray beam typically exhibits a bandwidth of Δ*E*/*E* ≃ 10^−4^, thereby discarding the majority of photons available within an undulator harmonic whose intrinsic bandwidth is of the order of 10^−2^. However, several important questions remain unresolved, including the extent of coherence degradation induced by finite energy bandwidth and the magnitude of chromatic aberration in broadband X-ray focusing systems (Ju *et al.*, 2018 ▸; Dufresne *et al.*, 2016 ▸). Accurate characterization of partially coherent pink X-ray beams is therefore essential for the design of efficient beamlines and for the quantitative interpretation of experimental data at modern facilities.

For rigorous coherence analysis, a wave-optics description is required. The wave-optics description of partially coherent X-ray beams from fourth-generation synchrotron radiation sources with ultra-low emittance is well established within the framework of cross-spectral density (CSD). However, wave-optics simulation of partially coherent pink X-ray beams presents significant computational challenges:

(i) In Monte Carlo based tools such as *Synchrotron Radiation Workshop* (*SRW*) (Chubar *et al.*, 2013 ▸) and *X-ray Tracing* (*XRT*) (Tack *et al.*, 2020 ▸), approximately 10^3^–10^5^ electrons are typically sampled. Each electron trajectory generates an individual X-ray wavefront, requiring repeated evaluation of synchrotron radiation emission. Although *SRW* provides physically rigorous models and can, in principle, treat pink X-ray beams by sampling photon energies across the bandwidth, such an approach becomes computationally prohibitive for routine beamline design and analysis.

(ii) An alternative strategy involves direct computation of the CSD function, as implemented in software packages such as *COherent Modes for SYnchrotron Light* (*COMSYL*) (Glass & Sanchez del Rio, 2017 ▸). This method performs coherent-mode decomposition of CSD, with computational complexity scaling as O(*N*^4^) for *N* spatial grid points in each transverse dimension. Although efficient for monochromatic beams, this approach becomes prohibitively expensive for pink-beam analysis, where CSD must be evaluated and diagonalized at multiple photon-energy points across the bandwidth.

(iii) Generating coherent modes from the wavefront matrix *A* instead of CSD using singular value decomposition (SVD) (Brandt, 2023 ▸) reduces the computational complexity from O(*N*^4^) to O(*nN*^2^), where *n* denotes the number of sampled electrons. Software packages such as *Coherent Analysis Toolbox* (*CAT*) (Xu *et al.*, 2022 ▸) and *XRT* employ this strategy. However, construction of the wavefront matrix *A* still requires explicit calculation of electron-generated wavefronts.

To address these challenges, we develop a computational framework that integrates three key components: firstly, a Monte Carlo Brightness Convolution (MCBC) method for efficient sampling of the electron phase space and construction of the wavefront matrix; secondly, a Hierarchical Incremental SVD (HI-SVD) strategy that alleviates the memory bottleneck of conventional approaches, enabling low-memory coherent-mode decomposition of the wavefront matrix *A*; and thirdly, a spectral–spatial decomposition that extracts a broadband, energy-dependent coherent-mode basis, thereby enabling rapid reconstruction of spatial coherence at arbitrary photon energies within the sampled bandwidth. This integrated framework enables efficient analysis of pink-beam coherence, which has previously been computationally prohibitive using conventional approaches. This paper presents the theoretical formulation, numerical implementation and validation of the proposed framework. We begin by outlining the theoretical foundations that combine partial-coherence theory and undulator radiation physics (Section 2), followed by a detailed description of the computational framework. The framework is subsequently benchmarked against *SRW* simulations and experimental measurements in Section 3. After establishing its reliability, Section 4 applies the framework to analyze chromatic focus degradation in compound refractive lens systems. The proposed framework therefore provides a practical tool for designing and optimizing experiments utilizing partially coherent, high-flux pink X-ray beams from fourth-generation light sources.

## 2. Theoretical framework

### 2.1. MCBC for wavefront matrix construction

According to the brightness convolution theory developed by Kim (1986 ▸), Geloni *et al.* (2008 ▸) and Glass & Sanchez del Rio (2017 ▸), the CSD function *W*(*r*_1_, *r*_2_, ω) at the virtual source of undulator can be expressed as
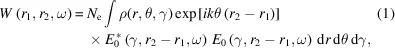
where *N*_e_ denotes the number of electrons per bunch, and ρ represents the five-dimensional electron phase space distribution, dependent on spatial coordinates *r*, angular divergence θ and the relativistic Lorentz factor γ (representing energy spread). This formulation relies on the assumption that electrons are statistically independent and that magnetic-field variations across the electron beam’s transverse dimensions are negligible. The reference electric field *E*_0_ is calculated using the Liénard–Wiechert formalism,
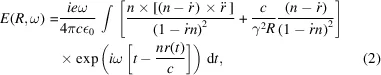
where *e* is the electron charge, *c* the speed of light, ε_0_ the electric constant, *n* is the unit vector pointing from the particle to the observation point *R*, *r*(*t*) is the electron trajectory of the reference electron. In the following MCBC formulation, this field is re-denoted as the reference wavefront *E*_0_(γ, *r*, ω), where *r* is the transverse coordinate on the wavefront plane, and ω is the sampled photon angular frequency.

The brightness convolution theory gives the relationship between the reference wavefront *E*_0_(γ, *r*, ω) and the wavefront generated by a sampled macro-electron (Chubar *et al.*, 2013 ▸) *E*_macro_(γ, *r*, ω),
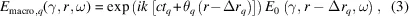
where Δ*r*_*q*_ and θ_*q*_ are the transverse offset and angular deviation of the *q*th macro-electron sampled from ρ(*r*, θ, γ) by Monte Carlo method (Chubar *et al.*, 2013 ▸). As discussed in the *Introduction*[Sec sec1], decomposing the full four-dimensional (4D) CSD matrix requires significant memory, and SVD of the wavefront matrix *A* necessitates Monte Carlo based wavefront calculations using equation (2). To address this, we employ an MCBC method to construct the wavefront matrix *A* based on the relationship derived from equation (3).

(i) *Energy-resolved reference wavefront calculation.* The electron energy spread is first discretized into a set of electron-energy slices γ_*k*_, with statistical weights determined by the ρ(*r*, θ, γ), typically over the range of ±σ_γ_. For each electron-energy slice γ_*k*_ at photon energy ω_*j*_, the reference wavefront with zero transverse offset and zero angular deviation *E*_0_(γ_*k*_, *r*, ω_*j*_) is calculated from the Liénard–Wiechert formalism in equation (2).

(ii) *Weighted phase-space sampling.* For each electron-energy slice γ_*k*_, a statistical ensemble of macro-electrons is generated by Monte Carlo sampling of the transverse electron position *r* and angular deviation θ from the corresponding electron phase-space distribution ρ(*r*, θ, γ). The number, or equivalently the statistical weight, of macro-electrons assigned to each energy slice is determined by ρ(*r*, θ, γ_*k*_). This step therefore incorporates both the transverse phase-space distribution and the electron-energy spread into the wavefront-matrix construction.

(iii) *MCBC wavefront transformation and matrix assembly.* For each sampled macro-electron, the corresponding wavefront *E*_macro_(γ_*k*_, *r*, ω_*j*_) is generated from the reference wavefront *E*_0_(γ_*k*_, *r*, ω_*j*_) using the transformed relation in equation (3). Thus, the computationally expensive Liénard–Wiechert integral in equation (2) does not need to be reevaluated for every macro-electron. Instead, wavefront matrix *A* is assembled by applying the MCBC transformation to the energy-resolved reference wavefronts.

Therefore, by rigorously exploiting the physical relationships inherent in brightness-convolution theory, the MCBC approach replaces the repeated Liénard–Wiechert integral with efficient geometric transformations of reference wavefronts, yielding orders-of-magnitude improvements in computational speed and memory efficiency.

### 2.2. Hierarchical incremental singular value decomposition (HI-SVD) for coherent mode decomposition

Coherent mode decomposition of the wavefront matrix *A* is achieved via SVD, which factorizes matrix *A* and reveals the orthogonal coherent modes
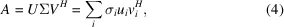
where *U* contains the spatial coherent modes *u*_*i*_(*r*, ω), Σ is a diagonal matrix of singular values σ_*i*_ (λ_*i*_ = 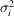 is the coherent faction of the *i*th coherent mode). Although the SVD of *A* avoids the O(*N*^4^) memory storage of full CSD, it still requires loading the entire O(*nN*^2^) matrix *A* in the memory- and time-consumption decomposition process. This becomes prohibitive for pink-beam analysis, where coherent-mode extraction must be repeated across multiple photon energies within the bandwidth.

To overcome this fundamental bottleneck, we employ a HI-SVD strategy. This approach adopts a Map–Reduce architecture (Khezr & Navimipour, 2017 ▸), transforming the decomposition of wavefront matrix *A* into a sequence of manageable low-rank subspace projections. In the Map phase we exploit the block-wise additivity of the CSD matrix. Synchrotron radiation from a storage ring is usually modeled as an incoherent superposition of wavefronts emitted by *n* independent electrons. Consequently, the CSD matrix satisfies the linear superposition (Hettel, 2014 ▸)
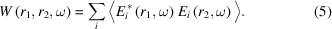
By partitioning the wavefronts into *s* batches, the CSD matrix can be represented as
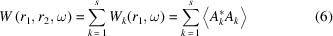
where
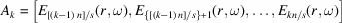
is the *k*th batch of wavefront matrix. The optimal low-rank approximation of matrix *A*_*k*_ can be calculated by a truncated SVD with a truncation rank *m*,
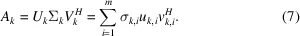
In the Reduce phase, the low-rank approximations from all batches are aggregated into reduced wavefront matrix *B* = [*A*_0_, *A*_1_, …, *A*_*s*_], which is subsequently decomposed to obtain the final coherent modes.

Compared with standard full SVD, the HI-SVD strategy offers significant advantages in both memory consumption and computational efficiency. Firstly, a standard SVD requires loading the entire wavefront matrix *A* into memory, with a space complexity of *O*(*nN*^2^). In contrast, the Map–Reduce strategy of HI-SVD requires memory only for a single batch *A*_*k*_ or for the aggregated low-rank approximation matrix *B*. Thus, the memory complexity of HI-SVD is reduced to*O*[*N*^2^ × max(*n*/*s*,* sm*)], which overcome the memory bottleneck of coherent mode decomposition using SVD. Secondly, the computational efficiency of standard SVD generally scales with *O*(*n*^2^*N*^2^). For HI-SVD method, the computational cost scales as *O*[*s* × *N*^2^ × (*n*/*s*)^2^] + *O*[*N*^2^ × (*sm*)^2^], corresponding to the Map and Reduce part of HI-SVD.

**Figure 1:**
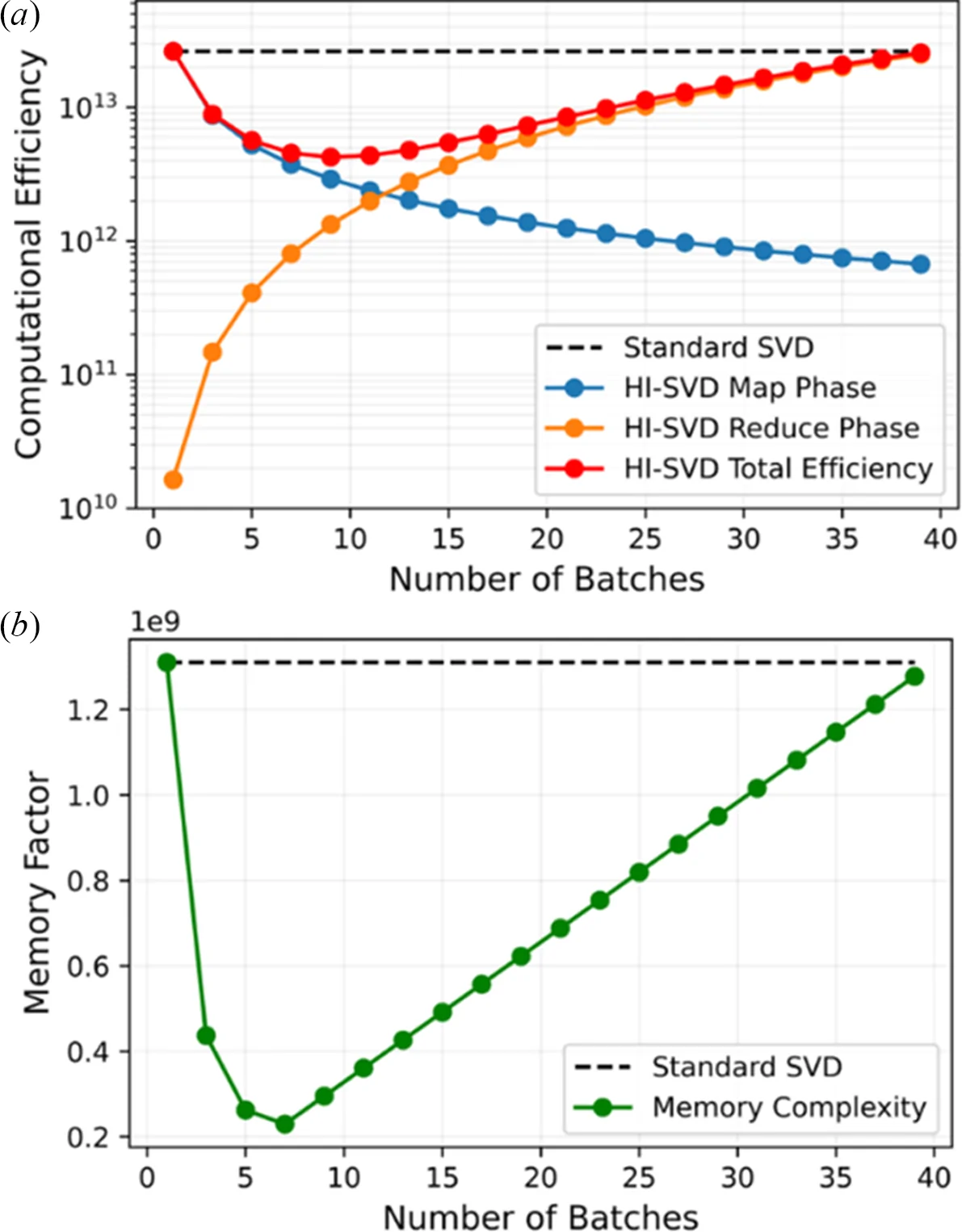
(*a*) Computational efficiency and (*b*) memory cost analysis of HI-SVD with the wavefront batch number *s*. Parameters: total number of wavefronts (electrons) *n* = 2 × 10^4^, a pixel resolution of *N* = 256^2^ and a truncation mode number *m* = 500.

As shown in Fig. 1 ▸, appropriate selection of the wavefront batch number *s* enables optimization of both memory consumption and computational cost by approximately one order of magnitude. It is worth noting that, although the Map phase can be executed in parallel to process multiple batches simultaneously, the typically small value of *s* limits the achievable level of parallelization. In the present framework, primary parallelization is performed across the spectral domain by distributing different photon energies within the pink-beam bandwidth. Specifically, photon-energy sampling is distributed across multiple computational nodes or CPU cores to maximize throughput. For distributed-memory systems, the message passing interface (MPI) (Walker & Dongarra, 1996 ▸) is employed, with each MPI rank assigned a subset of the photon-energy grid to process independently. Each rank executes the MCBC and HI-SVD pipeline for its assigned energy, ensuring that the *O*[*N*^2^×max(*n*/*s*,* sm*)] memory complexity is maintained on a per-node basis.

### 2.3. Spectral–spatial decomposition and propagation for pink-beam analysis

The combination of MCBC and HI-SVD yields a set of coherent modes (labeled *i*, range from 0 to *m* − 1) independently evaluated at *N*_E_ discrete photon energies (labeled *j*, range from 0 to *N*_E_ − 1) across the pink-beam bandwidth. While this approach rigorously captures the partial coherence at each energy, directly storing, propagating and analyzing these coherent modes *C* = {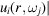
*i* = 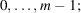

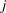
 = 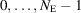} is computationally inefficient. To overcome this limitation, we introduce a spectral–spatial decomposition and propagation framework that explicitly separates the broadband coherence problem into a compact spatial basis and energy-dependent coefficients.

Instead of treating each energy as an independent wave-optics problem, the spectral–spatial decomposition extracts a global orthogonal spatial basis (labeled *l*, range from 0 to *m*′ − 1) that efficiently represents the coherent structure over the entire bandwidth. We therefore seek a separable representation of the coherent modes in the form
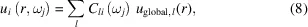
where *u*_global, *l*_(*r*) is a set of orthonormal spatial modes and *C*_*li*_(ω_*j*_) is an energy-dependent expansion coefficient. In practice, the global spatial modes are obtained by performing a singular value decomposition on the aggregated coherent modes (rooted-eigenvalue-weighted) matrix *C* from all sampled energies, retaining only the leading modes that capture the *m*′ dominant coherence structure. The principal advantage of this spectral–spatial decomposition is that it transforms the pink-beam coherence problem from a large set of independent monochromatic simulations into a low-rank representation that preserves both spatial coherence and spectral dependence.

Once the spectral–spatial decomposition is established, wave-optics propagation can be performed efficiently by propagating the global spatial modes *u*_global, *l*_(*r*) and reintroducing the energy dependence through dispersive propagation operators. For free-space propagation over a distance *z*, the Fresnel diffraction integral (Mendlovic *et al.*, 1997 ▸) for a given wavelength λ can be written in the spatial-frequency domain as
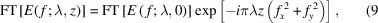
where *f* = (*f*_*x*_, *f*_*y*_) denotes the spatial frequencies and FT is the Fourier transform. For focusing optics such as compound refractive lenses (CRLs) (Dufresne *et al.*, 2016 ▸), chromatic effects arise from the energy dependence of the complex refractive index *n*(*E*) = 1 − δ(*E*) + *i*β(*E*), where δ(*E*) is the refractive-index decrement and β(*E*) describes absorption. The focal length of a CRL with *N* lenses and radius of curvature *R* is
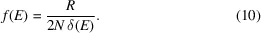
This expression shows that the CRL focal length is energy dependent through δ(*E*). Away from absorption edges, δ(*E*) approximately follows an *E*^−2^ dependence, leading to chromatic aberration for broadband pink-beam propagation.

To efficiently evaluate this dispersive propagation for all energies, the global spatial modes *u*_global, *l*_(*r*) is expanded represented as a three-dimensional tensor *u*_global, *l*_(ω, *y*, *x*), and all FT are performed in parallel on a GPU using CUDA acceleration (Farber, 2011 ▸). The propagated global mode *u*_global, *l*_(*r*, *z*) can be used to reconstruct the coherent mode *u*_*i*_(*r*, *z*, ω_*j*_) using *C*_*li*_(ω_*j*_)
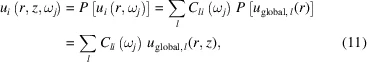
where *P* denotes propagation using a Fresnel propagator and interaction with optics. This approach avoids explicit loops over photon energy and enables the simultaneous propagation of all spectral components.

## 3. Application

The application presented in this section is based on the Hard X-ray Coherent Scattering (HXCS) beamline at HEPS. The HXCS beamline is designed for coherent X-ray scattering experiments and benefits from the ultra-low-emittance electron beam of HEPS, providing high transverse coherence in the hard X-ray regime. The undulator has a period length of 19.9 mm with 201 periods and operates at the first harmonic energy of 12.4 keV. The electron beam corresponds to a 6.0 GeV storage ring operating at a beam current of 0.2 A. The transverse phase-space distribution is characterized by r.m.s. beam sizes of 9.33 µm and 2.44 µm in the horizontal and vertical directions, respectively, together with r.m.s. angular divergences of 3.33 µrad (horizontal) and 1.28 µrad (vertical).

**Figure 2:**
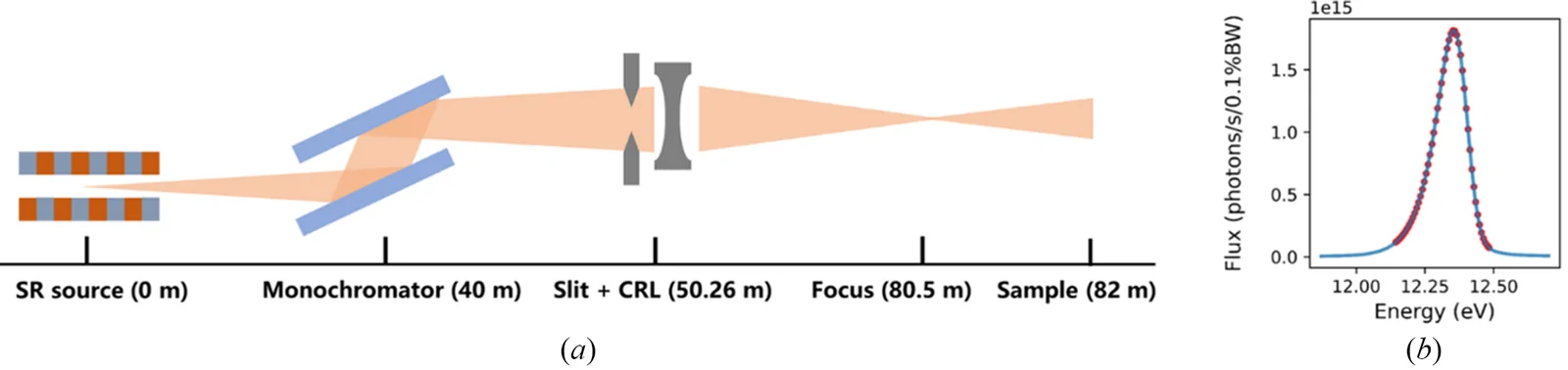
(*a*) Beamline layout illustrating the propagation of undulator radiation from the source through a multilayer monochromator operated in pink-beam mode, followed by a coherent slit and a CRL system, focused at 80.5 m and finally defocused at the experimental station. (*b*) Spectral flux distribution calculated by *SRW* code showing the finite bandwidth of the pink beam around the first harmonic and 50 discrete energy points were sampled around the first-harmonic peak.

In the current HXCS beamline design, a double-crystal monochromator is employed to deliver monochromatic radiation for coherence-based experiments. However, motivated by the partially coherent pink-beam simulation framework developed in this work, we investigate an alternative beamline operation mode by employing a multilayer monochromator operating in pink-beam mode. In this configuration, the radiation from the first harmonic of the undulator is selected with a finite energy bandwidth [Fig. 2 ▸(*b*)].

### 3.1. Single-energy source characterization using MCBC and HI-SVD

As the first step toward broadband pink-beam wave-optics simulations, we evaluate the performance of the proposed MCBC+HI-SVD for the coherent-mode analysis of a monochromatic undulator source (12.4 keV).

**Figure 3:**
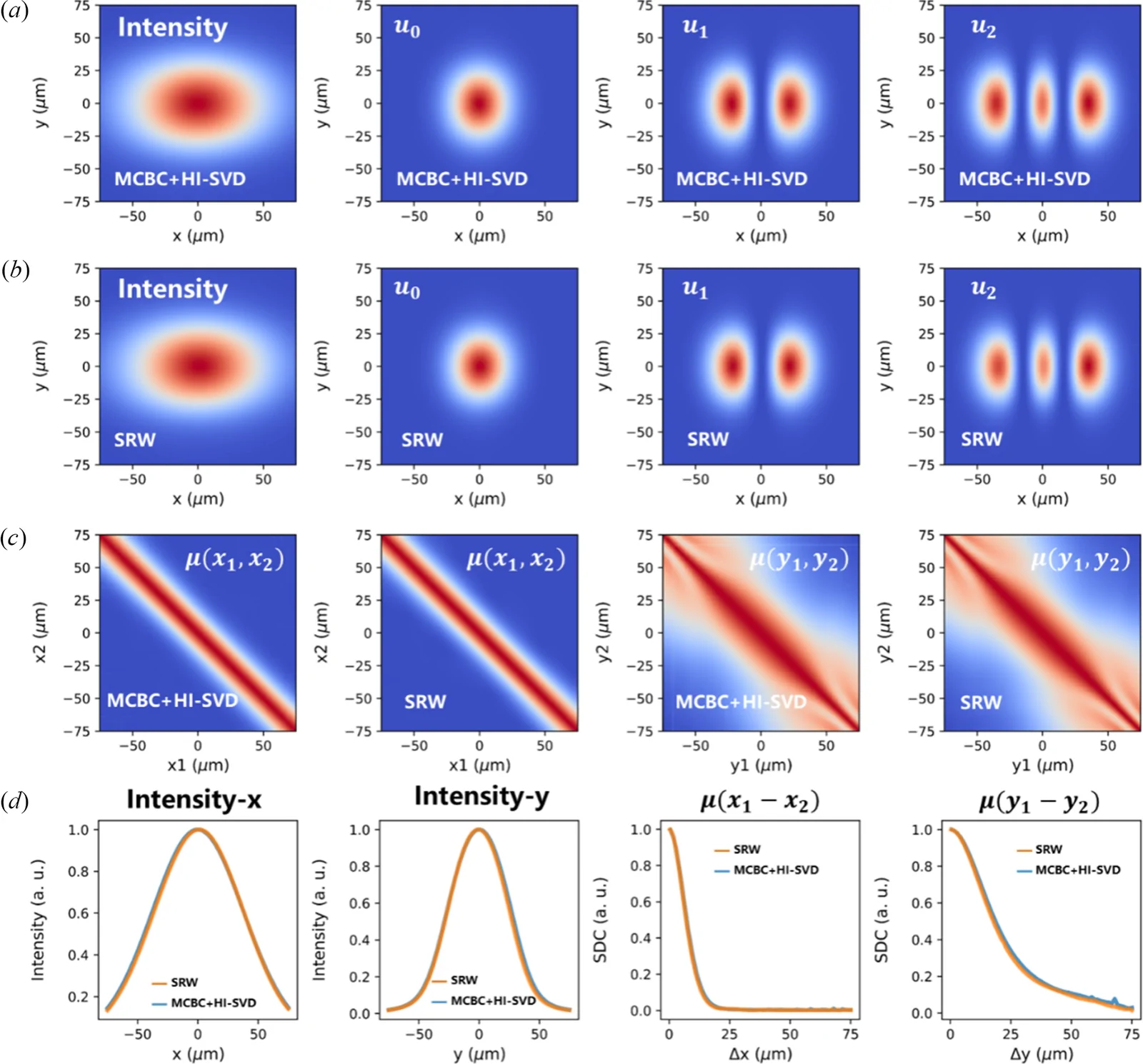
Single-energy coherent-mode characterization of the HEPS-B4 undulator source at 12.4 keV using the proposed MCBC+HI-SVD framework, with comparison to *SRW*. Total number of wavefronts (electrons) *n* = 2 × 10^4^, a pixel resolution of *N* = 256^2^, and a truncation mode number *m* = 500. (*a*) Transverse intensity distribution and the first three dominant coherent modes *u*_0_, *u*_1_, *u*_2_ obtained from MCBC+HI-SVD. (*b*) Corresponding results calculated independently with *SRW*, showing nearly identical intensity and modal structures. (*c*) DOC functions μ(*x*_1_, *x*_2_) and μ(*y*_1_, *y*_2_) evaluated in the horizontal and vertical directions, respectively, demonstrating excellent agreement between the two methods. (*d*) Quantitative comparison of the transverse intensity profiles and the coherence decay μ(*x*_1_ − *x*_2_) and μ(*y*_1_ − *y*_2_) as functions of spatial separation, confirming the high accuracy of MCBC+HI-SVD for coherent-mode decomposition at a single photon energy.

A major advantage of the MCBC+HI-SVD framework is its high computationally efficient and scalability. In the present benchmark, the complete coherent-mode decomposition was achieved within approximately 14 min on a personal computer. The peak memory consumption during the full simulation was only 5.09 GB, demonstrating a substantial reduction compared with conventional coherent mode decomposition approaches that typically require large-scale workstations or high-performance computing clusters for comparable coherent mode extraction. Fig. 3 ▸(*a*) presents the resulting intensity distribution, dominant coherent modes *u*_*i*_(*r*), and spectral density of degree (SDC) μ(*r*) obtained from the proposed method. The reconstructed intensity profile exhibits the expected Gaussian-like envelope typical of undulator radiation under realistic electron beam emittance. The first three coherent modes reveal the characteristic modal structure of partially coherent synchrotron radiation.

To further validate the physical accuracy of the MCBC+HI-SVD results, we performed a direct comparison with the widely used *SRW* code (Chubar *et al.*, 2013 ▸). The agreement of intensity, coherent modes [Fig. 3 ▸(*b*)] and SDC [Fig. 3 ▸(*c*)] between the two approaches is excellent. Both the transverse intensity profiles along the horizontal and vertical directions and the SDC functions μ(*x*_1_ − *x*_2_) and μ(*y*_1_ − *y*_2_) show nearly identical behavior. In particular, the coherence decay as a function of transverse separation is reproduced with high fidelity, confirming that the proposed method accurately captures the coherence properties of the source.

These results demonstrate that MCBC+HI-SVD provides an efficient and reliable approach for extracting coherent modes of synchrotron undulator radiation at a single photon energy. The combination of high numerical accuracy, low memory requirement, and fast runtime makes it particularly suitable as the computational foundation for broadband pink-beam simulations, where coherent mode decomposition must be performed repeatedly across multiple energies.

**Figure 4:**
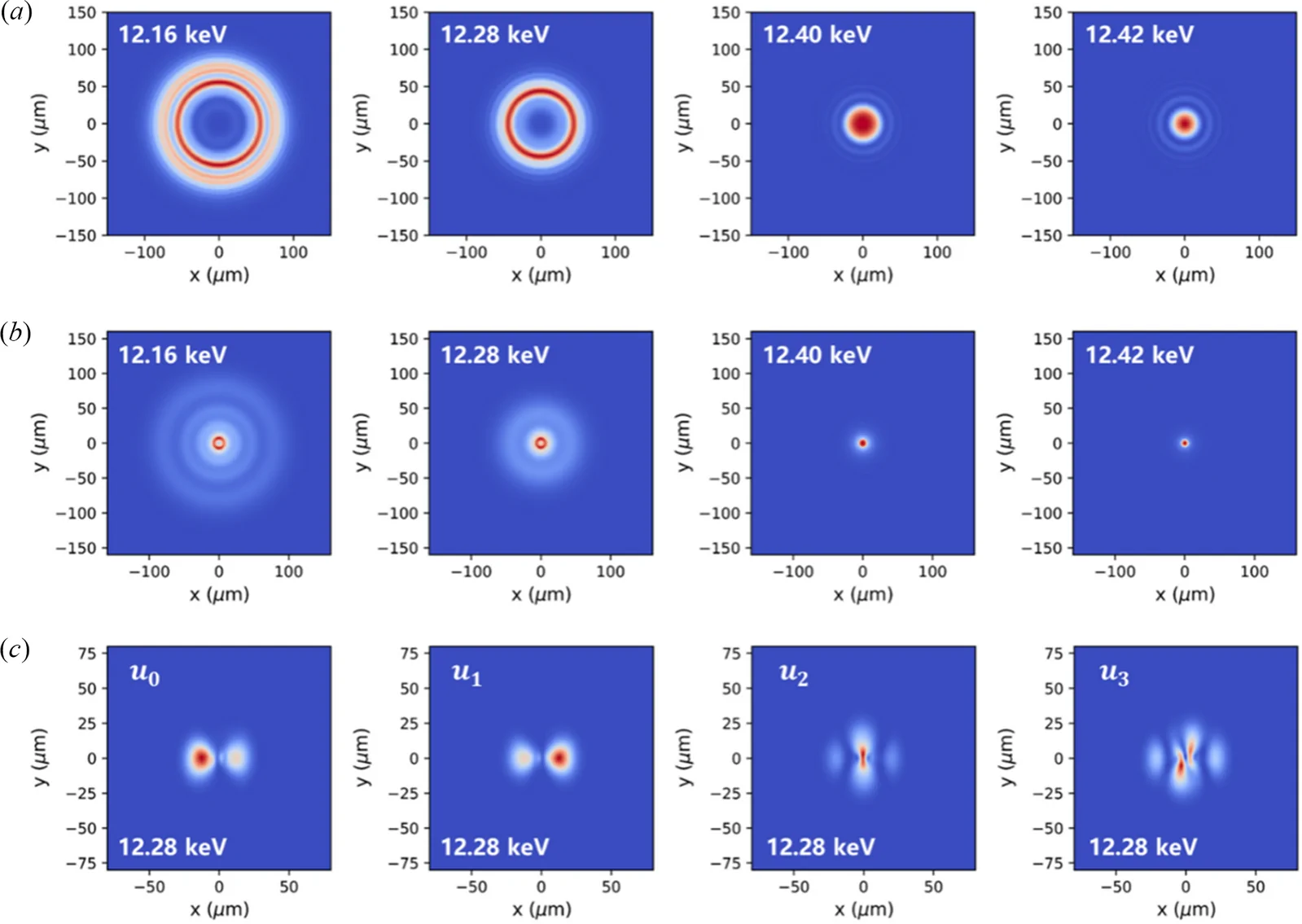
(*a*) Intensity distributions of the reference wavefronts *E*_0_(γ_0_, *r*, ω) calculated using *SRW* at four photon energies across the first undulator harmonic. (*b*) The wavefronts are back-propagated from the observation screen to the virtual source plane. (*c*) The first four dominant coherent modes *u*_*i*_(*r*) at 12.28 keV obtained using the proposed MCBC+HI-SVD framework.

Therefore, we extend the proposed MCBC+HI-SVD framework to the full broadband regime required for pink-beam simulations. In contrast to monochromatic coherence analysis, pink beams involve a finite energy bandwidth, such that coherent mode decomposition must be performed across multiple photon energies while accounting for chromatic wavefront evolution. As shown in Fig. 4 ▸(*a*), the reference wavefronts *E*_0_(γ_0_, *r*, ω) across the pink-beam bandwidth are calculated and back propagated to the virtual source [Fig. 4 ▸(*b*)]. By employing the MCBC method, the wavefront matrix at each energy was constructed and further decomposed using the HI-SVD algorithm, yielding a compact set of coherent modes *u*_*i*_(*r*) and eigenvalues [Fig. 4 ▸(*c*)].

Each photon energy represents a statistically independent monochromatic slice, which is suitable for parallel calculation. The parallel computations were performed on a Linux-based high-performance computing server running under KVM virtualization. The system was equipped with 50 physical CPU cores (Intel Xeon Ice Lake architecture, single-threaded per core) and a total system memory of approximately 250 GB. The computation was parallelized over 50 CPU cores, and the complete computation was achieved within approximately 71 min.

### 3.2. Broadband pink-beam coherent-mode analysis and spectral–spatial decomposition

**Figure 5:**
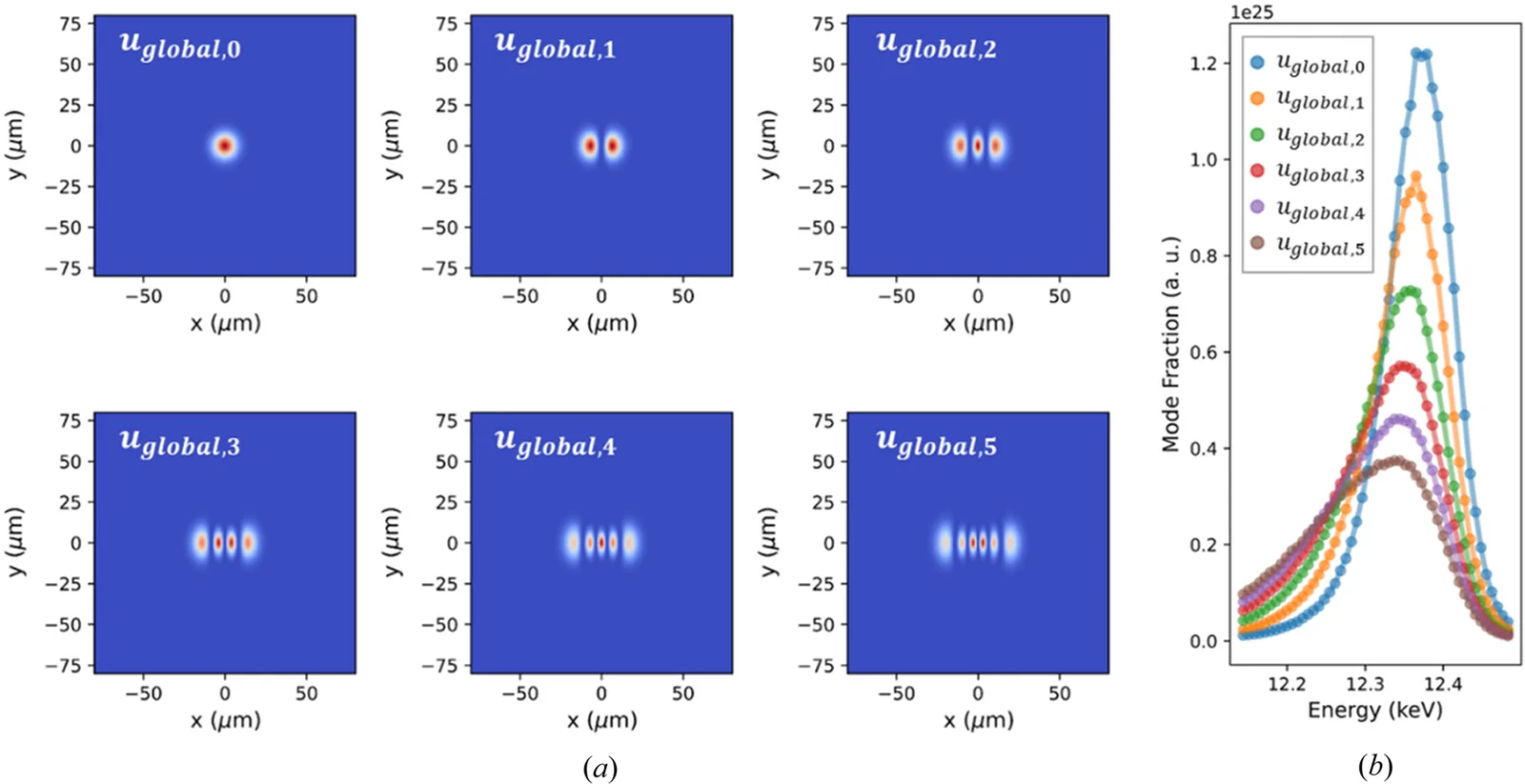
(*a*) The first six spectral–spatial modes *u*_global, *l*_(*r*). (*b*) Mode fractions of the spectral–spatial modes as a function of photon energy, showing the energy dependence of each mode’s contribution to the overall coherence structure of the source. The results demonstrate the efficient representation of the undulator radiation’s coherence properties across the entire energy range.

All energy-resolved coherent modes *C* = 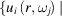
*i* = 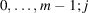 = 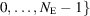 were aggregated and used to perform a spectral–spatial decomposition. In this approach, the energy dependence is separated from the dominant spatial degrees of freedom by extracting a set of global orthonormal spatial basis functions, referred to as spectral–spatial modes *u*_global, *l*_(*r*) [Fig. 5 ▸(*a*)]. These global modes form an efficient broadband spatial basis that captures the dominant coherence structure of the undulator source over the entire pink-beam bandwidth [Fig. 5 ▸(*b*)].

**Figure 6:**
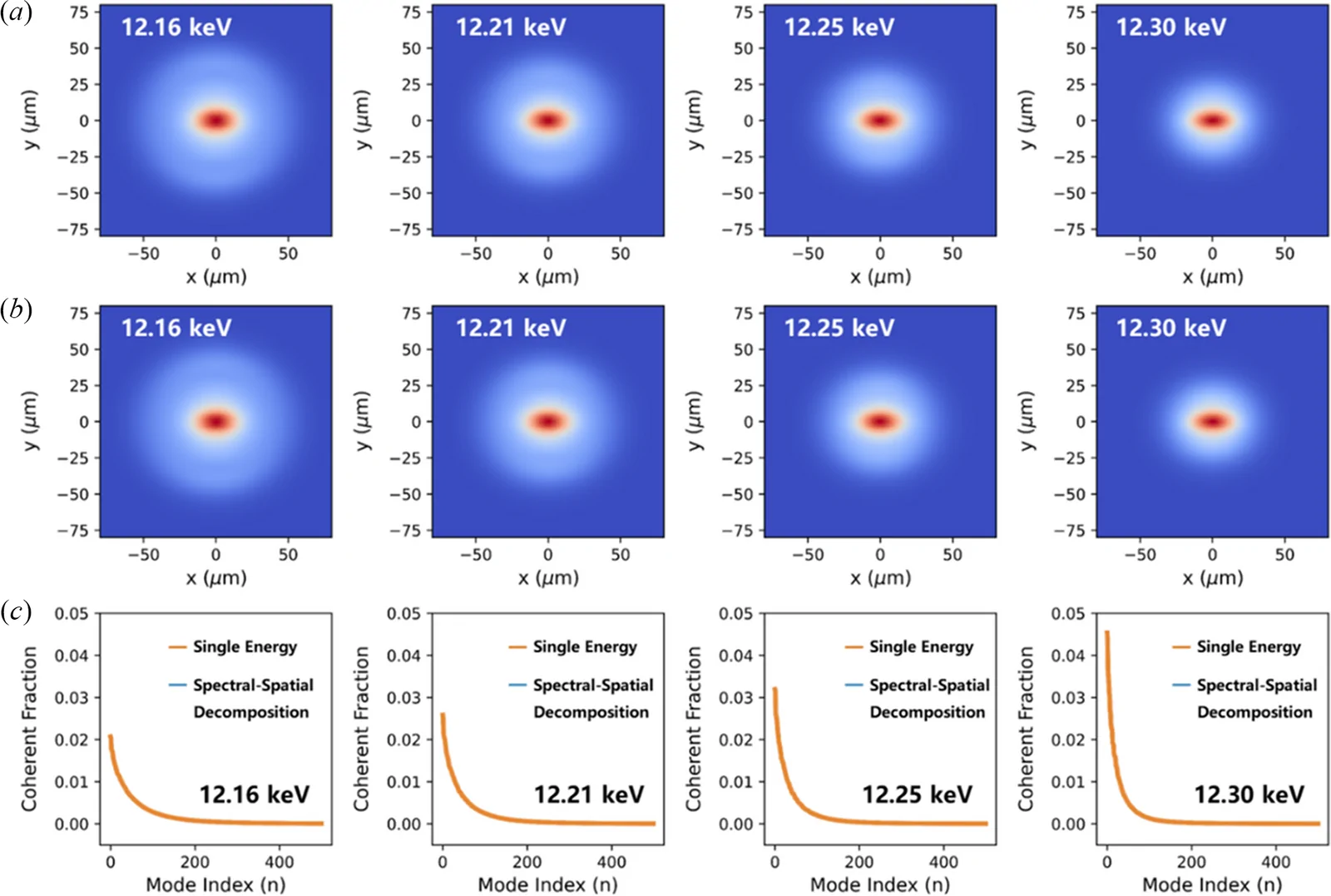
(*a*) Intensity distributions reconstructed at four representative photon energies using the propagated spectral–spatial modes and the corresponding energy-dependent expansion coefficients. (*b*) Intensity distributions obtained from independent coherent-mode decompositions performed directly at each sampled photon energy. (*c*) Comparison of coherent fractions as a function of mode index for the reconstructed modes (spectral–spatial decomposition) and the independently computed single-energy modes at the same photon energies.

After obtaining the spectral–spatial modes *u*_global, *l*_(*r*), the broadband wave-optics propagation can be performed by propagating only the spectral–spatial modes (detailed in Section 2.3), followed by energy-dependent reconstruction of the coherent modes using *C*_*li*_(ω_*j*_). To verify the accuracy of this reconstruction, we compared the intensity distributions and coherent fractions obtained from reconstructed energy-dependent modes with those computed directly from independent sampled energy. As shown in Fig. 6 ▸, both the transverse intensity profiles [Figs. 6 ▸(*a*) and 6 ▸(*b*)] and the coherent fraction [Fig. 6 ▸(*c*)] overlap perfectly, confirming that the spectral–spatial decomposition preserves the full coherence information across the bandwidth without loss of accuracy.

Overall, these results demonstrate that the proposed MCBC+HI-SVD method, and spectral–spatial decomposition, propagation and reconstruction, provide a computational efficiency and physically rigorous framework for pink-beam coherence simulations. By reducing the broadband problem to the propagation of a limited set of global spatial modes, the method enables efficient wave-optics modeling of chromatic coherent analysis in synchrotron radiation beamlines.

### 3.3. Broadband pink-beam coherent-mode analysis and spectral–spatial decomposition and propagation

**Figure 7:**
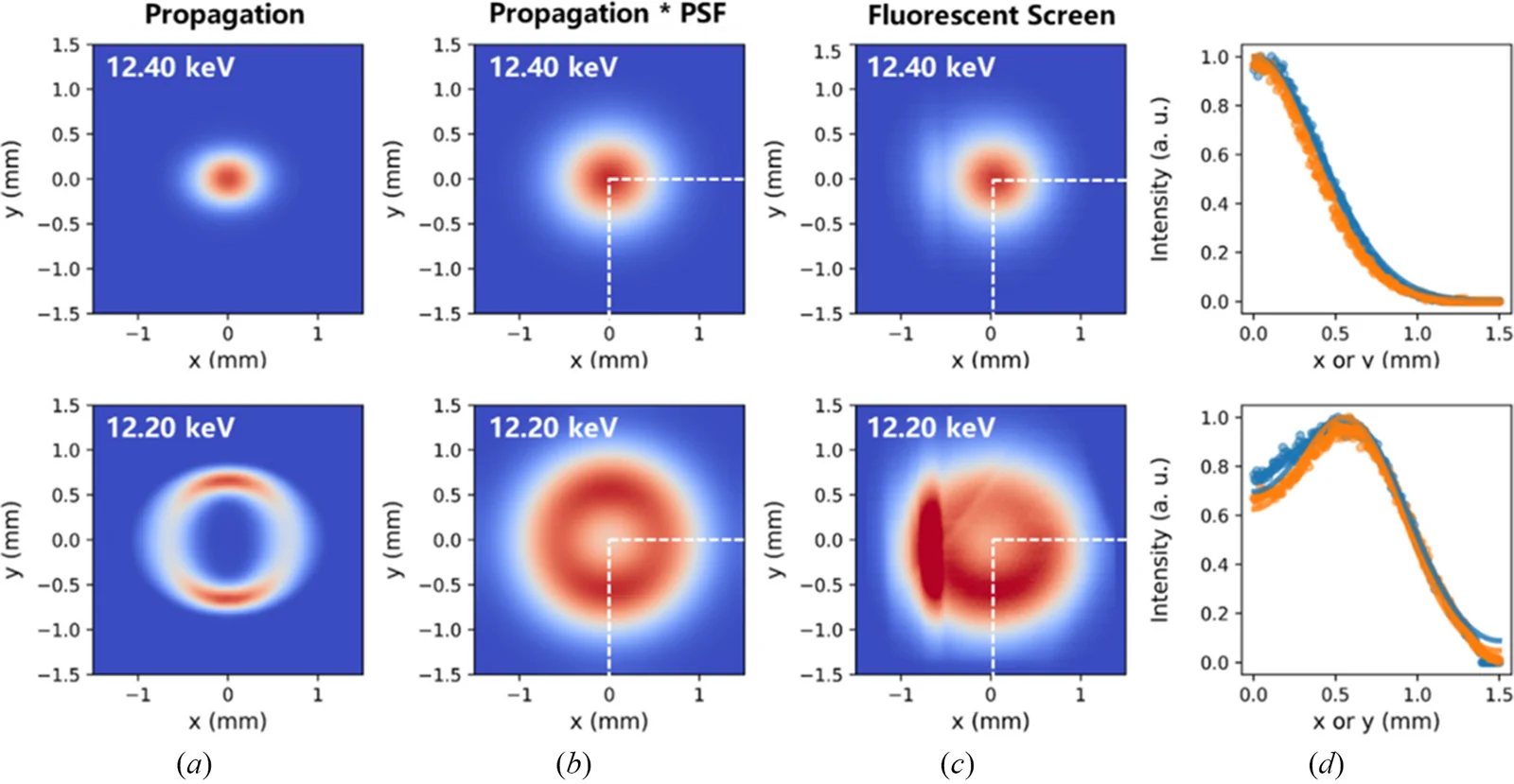
Validation of spectral–spatial mode propagation against experimental measurements at the HEPS HXCS beamline. (*a*) Simulated intensity distributions reconstructed from propagated modes at photon energies of 12.40 keV and 12.20 keV. (*b*) Simulated intensities after convolution with a Gaussian point-spread function to account for the finite resolution of the imaging system. (*c*) Experimentally measured fluorescence images acquired at 51.3 m from the source. Note that the non-uniform intensity features observed on the left side of the image correspond to the marks on the fluorescence screen. (*d*) Quantitative comparison of horizontal and vertical line profiles extracted from the beam center. Blue dots and orange dots represent the experimental data, respectively, while the solid lines of the corresponding colors denote the PSF-convolved simulation results.

Firstly, we benchmarked the simulated intensity distributions against experimental measurements acquired at HEPS HXCS beamline configuration to validate the physical fidelity of the propagated spectral–spatial modes. In the experiment, a double-crystal monochromator (40 m) was used, and a fluorescence screen was installed at a position of 51.3 m from the source. Two representative photon energies were measured: 12.40 keV (central core) and 12.20 keV (red-shifted). In the simulation, the complex wavefield at the fluorescence screen plane was reconstructed from the propagated spectral–spatial modes (Section 2.3) and the corresponding energy-dependent coefficients *C*_*li*_(ω_*j*_). The resulting intensity distribution, *I*_sim_, is shown in the first column of Fig. 7 ▸(*a*). Since the measured fluorescence image is affected by the finite spatial resolution of the screen and imaging system, we modeled the detection blur by a Gaussian point-spread function (PSF) *I*_convolved_ = *I*_sim_*G_PSF_, where G_PSF_ is the 2D Gaussian PSF with standard deviation (σ_PSF_ = 0.31 mm) determined from the calibrated screen response. The PSF-convolved results are shown in Fig. 7 ▸(*b*), while the experimentally measured fluorescence images are shown in Fig. 7 ▸(*c*). A quantitative comparison is provided in Fig. 7 ▸(*d*), where horizontal and vertical line profiles extracted through the beam center are plotted for the PSF-convolved simulation, and the experimental measurement. For both energies (12.40 keV and 12.20 keV), the agreement of both the horizontal and vertical line profiles demonstrates that the spectral–spatial decomposition and propagation preserve the correct wave-optics propagation behavior.

**Figure 8:**
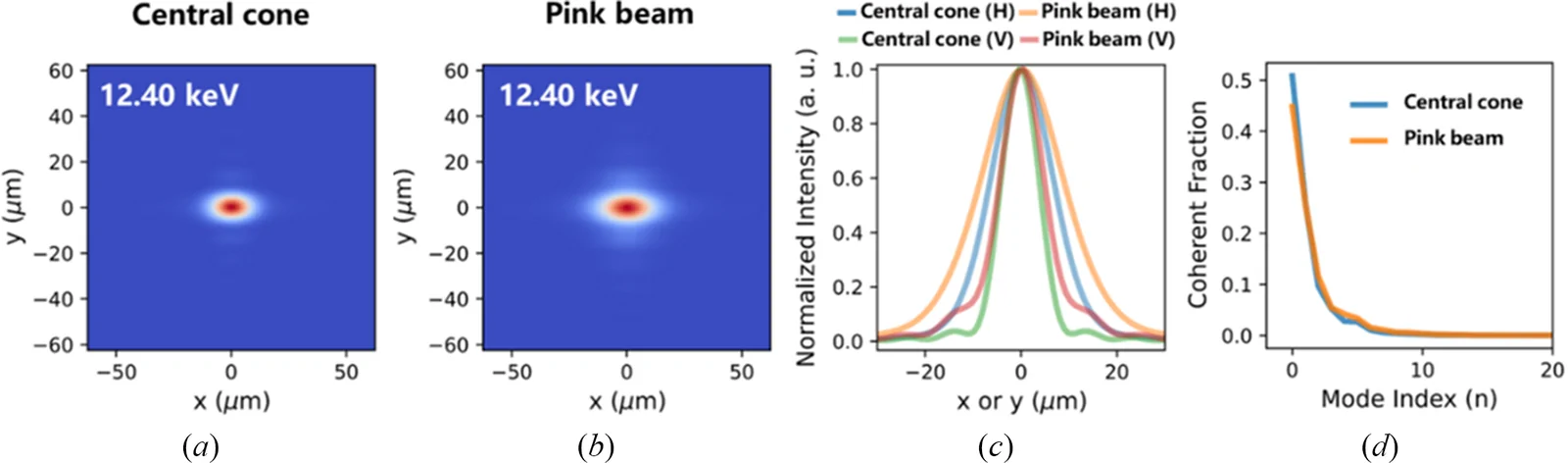
Comparison of the focus intensity and coherence properties for the quasi-monochromatic central cone and the broadband pink-beam cases at 12.40 keV under identical CRL focusing conditions. (*a*) Simulated 2D focal intensity map for the central cone. (*b*) Simulated 2D focal intensity map for the pink beam. (*c*) Normalized intensity profiles along the horizontal (H) and vertical (V) directions for both cases, showing the broadband-induced spot broadening in the pink-beam operation. (*d*) Coherent fraction of mode.

Using the broadband coherent-mode analysis and spectral–spatial decomposition developed in this work, we evaluated both the focus spot size and coherence for the quasi-monochromatic central cone and the broadband pink-beam case under identical CRL focusing conditions [Fig. 8 ▸]. At 12.4 keV, the central cone produces a compact focus with FWHM 15.6 µm × 9.0 µm, whereas the pink beam broadens to 19.8 µm×11.2 µm. This trend is consistent with the analytical chromatic aberration model (Dufresne *et al.*, 2016 ▸) for CRLs, where the energy dependence of the refractive-index decrement δ ∝ *E*^−2^ makes the focal length energy dependent and the broadband focus can be written as
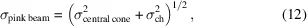
where σ_pink beam_ and σ_central cone_ are the Gaussian σ of focus size belong to pink beam and central cone. σ_ch_ is the chromatic blur term
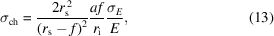
where *r*_s_, *r*_i_ and *f* are the source–lens distance (50.26 m), lens–focus distance (30.24 m) and focal length (18.63 m), respectively; σ_*E*_/*E* is the bandwidth of the pink beam (0.85%); *a* is the coherent slit half-size. For a 300 µm coherent slit size, the σ_ch_ is calculated as 3.97 µm, and the corresponding theoretical pink-beam focus size is 18.2 µm × 12.30 µm. The simulated pink-beam values (19.8 µm × 11.2 µm) are therefore in agreement with the theory at the level expected for an approximate Gaussian-kernel description. In addition, the coherent fraction decreases from 50.84% (central cone) to 44.83% (pink beam). Therefore, beyond reproducing the spot-size broadening, our framework explicitly propagates the broadband coherent modes and quantifies the coherence degradation at the focus, enabling a direct evaluation of the coherent fraction and mode-weight redistribution for pink-beam operation. As an additional example of an optical element with a strongly energy-dependent response, we simulated the propagation of a pink beam reflected by a grazing-incidence plane mirror; the details are provided in Section S1 of the supporting information.

## 4. Conclusion

We have developed and demonstrated a physically rigorous and computationally efficient wave-optics framework for simulating the transport of partially coherent pink X-ray beams. By integrating the MCBC method, HI-SVD strategy, we have effectively performed wavefront matrix construction and coherent mode decomposition. The introduction of spectral–spatial decomposition further extends this capability to the broadband regime by separating energy-dependent coefficients from a compact global spatial basis. This allows for the simultaneous propagation of all spectral components and the accurate reconstruction of spatial coherence at any energy within the spectrum. Our benchmarks against experimental data at the HEPS HXCS beamline confirm that the framework accurately captures wave-optics propagation behavior and chromatic effects. Specifically, the analysis of CRL focusing systems reveals the trade-offs between increased photon flux and the resulting coherence degradation (50.84% to 44.83%) and focus spot broadening in pink-beam modes. Therefore, this framework provides a fast and accurate means to design and interpret pink-beam coherent X-ray experiments.

## Supplementary Material

Section S1: energy-dependent filtering by a grazing-incidence plane mirror. DOI: 10.1107/S1600577526007034/tv5091sup1.pdf

## Data Availability

Data underlying the results presented in this paper are not publicly available at this time but may be obtained from the authors upon reasonable request.
