# Supplementary material for: An efficient wave-optics framework for partially coherent pink X-ray beam simulation and analysis
Source: J Synchrotron Radiat. 2026 Aug 31;33(Pt 5):1372–81. doi: 10.1107/S1600577526007034 (PMC13549356; doi:10.1107/S1600577526007034)
Supplement: Supplementary file 1 [file s-33-01372-sup1.pdf]

JOURNAL OF SYNCHROTRON RADIATION

Volume 33 (2026)

Supporting information for article:

An efficient wave-optics framework for partially coherent pink X-ray beam simulation and analysis

Shuo Wang, Han Xu, Liang Zhou and Ming Li

S1. Energy-dependent filtering by a grazing-incidence plane mirror

To further examine the behavior of the proposed spectral–spatial propagation framework for optical elements with strong energy-dependent responses, we considered a grazing-incidence plane mirror in a pink-beam configuration. The calculation geometry is shown schematically in Fig. S1(a). The pink X-ray beam emitted from the synchrotron-radiation source was propagated to a plane mirror located 10 m downstream from the source. After reflection, the beam was propagated by an additional 1 m to a screen.

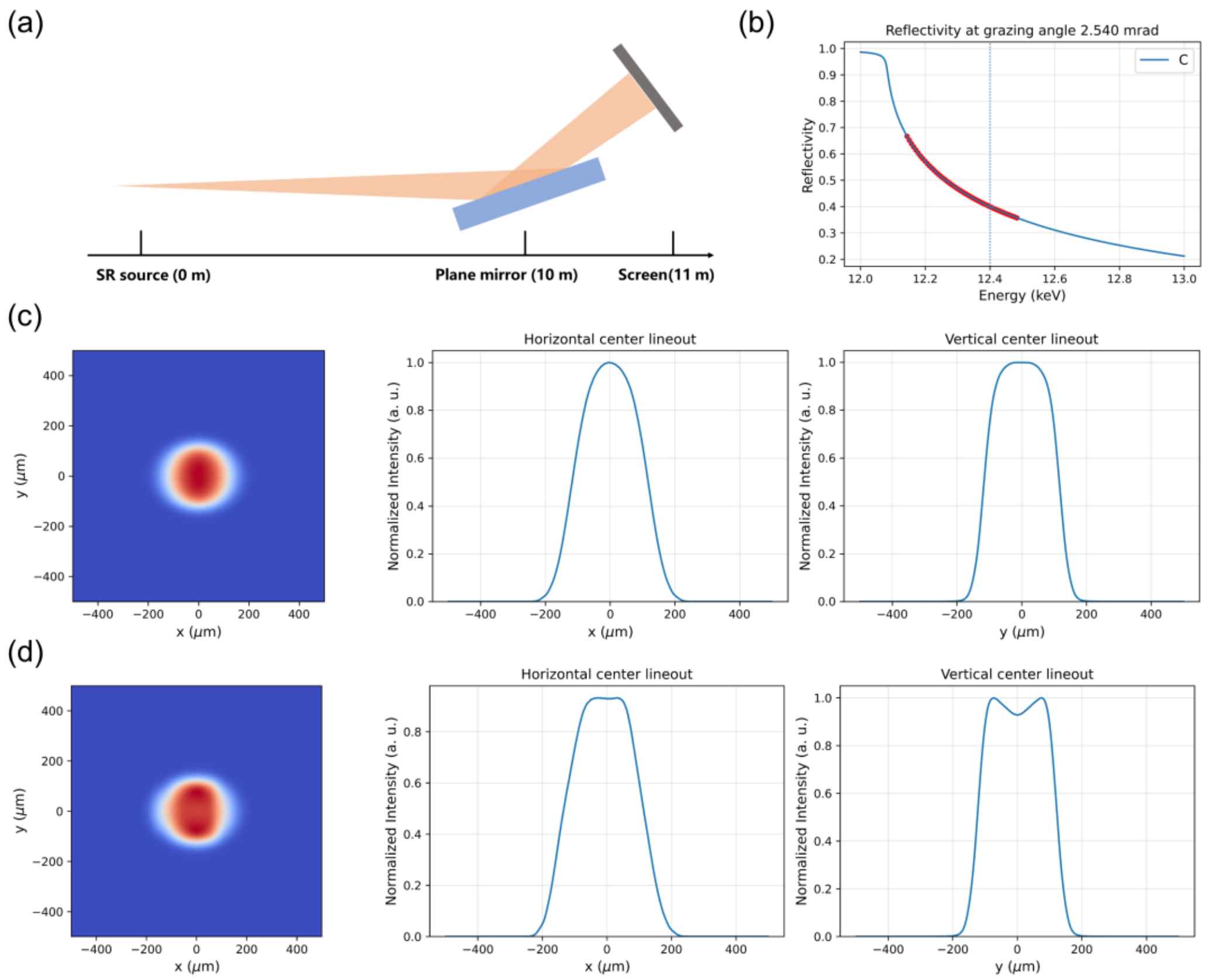

Figure S1 Energy-dependent filtering of a pink beam by a grazing-incidence plane mirror. (a) Schematic of the simulation model (Top view). (b) Calculated reflectivity of the carbon-coated plane mirror at the nominal grazing angle of 2.54 mrad. The red markers indicate the sampled photon energies used in the pink-beam source. (c) Intensity distribution and horizontal/vertical center lineouts without the energy-dependent mirror filtering effect. (d) Intensity distribution and corresponding center lineouts after applying the mirror response.

The plane mirror was coated with carbon and operated at a grazing angle of θ_0=2.54 mrad. The complex refractive index of the mirror coating was written as $n(E) = 1 - \delta(E) + i\beta(E)$, where $\delta(E)$ is the refractive-index decrement and $\beta(E)$ describes absorption. For each sampled photon energy, the sigma-polarized Fresnel reflection coefficient was calculated as

$$r_\sigma(E, \theta_0) = \frac{sin(\theta_0) - q(E, \theta_0)}{sin(\theta_0) + q(E, \theta_0)}$$

with $q(E, \theta_0) = \sqrt{n^2(E) - cos^2(\theta_0)}$. For an incident X-ray beam with finite angular spread, different angular components interact with the mirror at slightly different effective grazing angles. In the mirror incidence plane, the effective grazing angle can be expressed as

$$\theta = \theta_0 + \alpha(p_x, p_y)$$

where α is the angular deviation of the incident wavefront from the nominal optical axis of plane mirror, and (p_x, p_y) is the position at the plane mirror. Therefore, the mirror response was written as

$$r_\sigma(E, \theta) = r_\sigma[E, \theta_0 + \alpha(p_x, p_y)]$$

Figure S1(b) shows the calculated reflectivity of the carbon-coated plane mirror at the nominal grazing angle. The sampled pink-beam energies lie on a rapidly varying part of the reflectivity curve. In this energy range, the high-energy components are reflected less efficiently than the low-energy components.

According to Equation 11, the operator P denotes propagation using Fresnel propagator and interaction with optics elements. Thus, for the coherent mode $u_i(r, \omega_j)$ after the interaction with plane mirror, $r_\sigma[u_i(r, \omega_j)] = \sum_l C_{li}(\omega_j) r_\sigma[u_{global,l}(r)]$, where r_σ is the sigma-polarized Fresnel reflection coefficient, $u_{global,l}(r)$ is the global mode. Without the energy-dependent mirror response, as shown in Fig. S1(c), the propagated pink beam remains symmetric at the receiver screen. After the mirror response is included, the intensity distribution is significantly modified, as shown in Fig. S1(d). Because the carbon mirror suppresses the high-energy components more strongly, the reflected beam becomes dominated by the lower-energy part of the spectrum and produces a ring-like structure in the intensity distribution. In addition, the horizontal center lineout becomes asymmetric, which arises from the angular dependence of the grazing-incidence reflection.
